# Differential Interactions of Chiral Nanocapsules with DNA

**DOI:** 10.3390/ijms22020584

**Published:** 2021-01-08

**Authors:** Amani Zoabi, Katherine Margulis

**Affiliations:** The Institute for Drug Research, The School of Pharmacy, Faculty of Medicine, The Hebrew University of Jerusalem, Jerusalem 9112192, Israel; dr.amani.zoabi@gmail.com

**Keywords:** chiral nanoparticles, chiral polymeric nanocapsules, polyurea, stereospecific interactions, DNA binding

## Abstract

(1) Background: Chiral nanoparticular systems have recently emerged as a compelling platform for investigating stereospecific behavior at the nanoscopic level. We describe chiroselective supramolecular interactions that occur between DNA oligonucleotides and chiral polyurea nanocapsules. (2) Methods: We employ interfacial polyaddition reactions between toluene 2,4-diisocyanate and lysine enantiomers that occur in volatile oil-in-water nanoemulsions to synthesize hollow, solvent-free capsules with average sizes of approximately 300 nm and neutral surface potential. (3) Results: The resultant nanocapsules exhibit chiroptical activity and interact differentially with single stranded DNA oligonucleotides despite the lack of surface charge and, thus, the absence of significant electrostatic interactions. Preferential binding of DNA on d-polyurea nanocapsules compared to their l-counterparts is demonstrated by a fourfold increase in capsule size, a 50% higher rise in the absolute value of negative zeta potential (ζ-potential), and a three times lower free DNA concentration after equilibration with the excess of DNA. (4) Conclusions: We infer that the chirality of the novel polymeric nanocapsules affects their supramolecular interactions with DNA, possibly through modification of the surface morphology. These interactions can be exploited when developing carriers for gene therapy and theranostics. The resultant constructs are expected to be highly biocompatible due to their neutral potential and biodegradability of polyurea shells.

## 1. Introduction

Chiral nanomaterials have attracted considerable attention in recent years because they provide a powerful platform for exploring how chiral behavior is impacted by other unique properties attributed to nanometric size, and vice versa [1]. For example, the chiroptical activity can be modified and enhanced by controlling the size and the shape of nanoparticles stabilized by chiral biomolecules [2,3].

In the field of medical applications, chiral nanoparticles are emerging as true game changers owing to their apparent ability to uniquely interfere with various biological systems in vivo. Despite the prevalence of chirality in biology, exploiting chiral supramolecular assembly structures to achieve specific in vivo interactions and to facilitate drug delivery has only recently started being explored [4]. Thus, in early 2020, it was reported that the chirality of supraparticles created through a self-limiting assembly of cobalt oxide nanostructures capped with l-, or d-cysteine controls: (a) the binding of the nanoparticles to cell membrane lipids, (b) the internalization of the nanoparticles by cells and (c) their stability in human plasma [4]. Another recent study reported on a chirality-controlled ability of β-glucan nanoparticles to activate macrophages and produce immune enhancing cytokines which, in combination with their high antitumor drug loading, may enhance antitumor activity [5]. During 2019, chiral mesoporous silica nano-cocoon structures were demonstrated to enhance the aqueous solubility of their poorly-soluble drug cargo by converting it into its amorphous form [6]. Stereospecific induction of cancer cell death through apoptosis [4] or autophagy [7] also was reported for chiral nanoparticles in recent years.

In this study we synthesized chiral polymeric nanocapsules of polyurea using volatile nanoemulsion as the template for interfacial polymerization and studied their supramolecular interactions with DNA. Polyurea nanocapsules are biocompatible and biodegradable, their formation process is easily scalable, and they allow for the controlled delivery of a wide variety of drugs [8,9,10,11]. They were previously shown to protect drugs from premature degradation in vivo and enable targeted delivery [9]. However, the synthesis of chiral polymeric nanocapsules and, especially, of polyurea nanocapsules is not common; the first work was reported in 2018 by Zoabi et al., which described a successful synthesis of chiral ethyl cinnamate-containing polyurea nanocapsules by an interfacial cross-linking reaction using l-lysine and polymethylene polyphenylisocyanate. This system was demonstrated to induce chirality within liquid crystals [12]. Herein, we describe the stereospecific interactions of solvent-free chiral polyurea nanocapsules, synthesized by an interfacial polyaddition reaction between toluene 2,4-diisocyanate and lysine enantiomers, and single stranded DNA (ssDNA).

DNA is a key chemical structure that encodes biological information for regulation of all cellular functions. Targeted delivery of DNA presents one of the most promising directions in modern therapeutics. Thus, oligonucleotides (short single stranded DNA sequences) are being increasingly employed as the agents for targeted gene correction or for gene/mRNA silencing (antigen/antisense gene therapy) [13,14]. In vivo delivery of oligonucleotides is carried out by two major types of drug delivery systems (DDS): (1) Viral DDS based on nonpathogenic attenuated viruses and (2) Nonviral DDS based on cationic nanoparticles that form complexes with negatively charged DNA [14]. DDS based on viral vectors are known to cause a substantial immune response in some patients [15], while cationic nanoconstructs exhibit a significantly higher cytotoxicity in vivo compared with their anionic or neutral counterparts [16].

Here we report on the formation of chiral solvent-free and charge-free nanocapsules that have stereospecific interactions with ssDNA, rather than binding based on electrostatic interactions that occurs in cationic nanostructures. We infer that such stereospecific constructs may be employed as DDS for antigen/antisense gene therapy with a low potential for toxicity.

## 2. Results

### 2.1. Chiral Nanocapsules Formation and Characterization

**Scheme 1:**
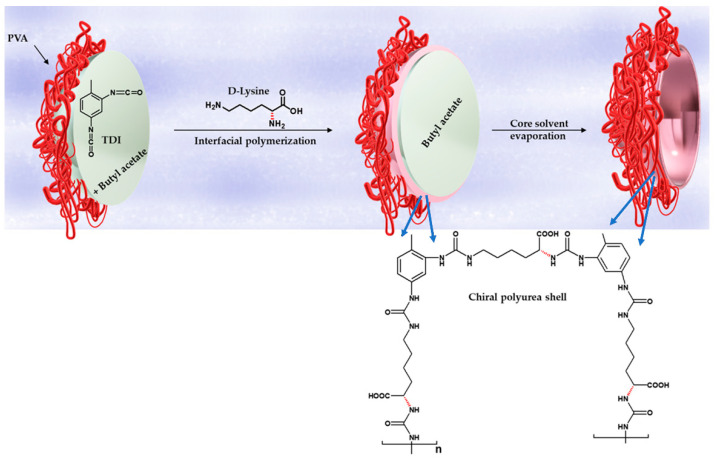
Preparation of d-polyurea nanocapsules. Oil-in-water nanoemulsion, stabilized by polyvinyl alcohol (PVA) and containing a toluene 2,4-diisocyanate (TDI) monomer dissolved in *n-*butyl acetate as the oil phase, is formed first. d-lysine then is added to the aqueous phase of this nanoemulsion, and the polyaddition reaction is initiated upon the diffusion of both TDI and d-lysine to the droplet interface. Concomitant polymerization and core solvent evaporation continue until the formation of hollow, solvent-free nanocapsules is completed.

The chiral capsules in the submicron size range (further referred to as “nanocapsules”) were prepared by interfacial polymerization in emulsion followed by volatile solvent evaporation. The chirality was controlled by the addition of two enantiomers of lysine amino acid to the capsule shell. l- or d-lysine was introduced to the oil-in-water emulsion system containing toluene 2,4-diisocyanate (TDI) monomers dissolved in *n-*butyl acetate as the organic phase, and distilled water with dissolved polyvinyl alcohol (PVA, nonionic polymeric surfactant) as the aqueous phase. Ultrahigh sonication was first applied to the system to disperse the organic phase as nanometric droplets. Either an l- or a d-lysine enantiomer was then added to this system, and an interfacial polyaddition reaction occurred upon the encounter between oil-soluble TDI monomers and water-soluble lysine at the oil droplet interface. The system was kept under slight heating at 35 °C overnight to evaporate the volatile solvent and to form the final chiral d- or l-polyurea nanocapsules. The synthesis of d-polyurea nanocapsules is depicted in Scheme 1. The same synthesis procedure using the l-lysine enantiomer led to the formation of l-polyurea nanocapsules.

**Figure 1:**
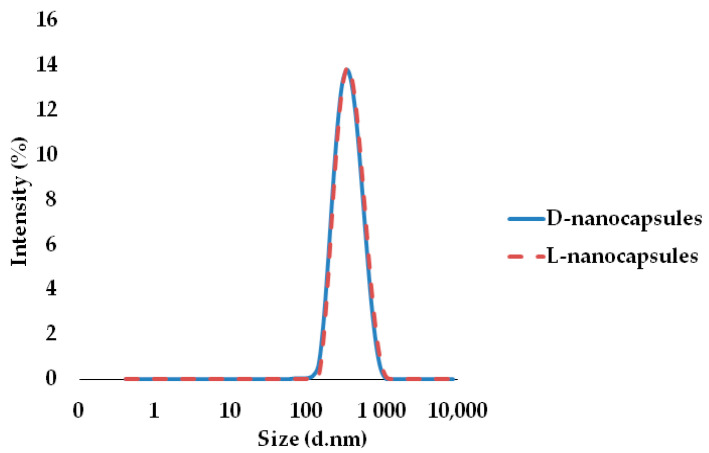
Representative size distributions by intensity of: d-polyurea nanocapsules (blue, solid) and l-polyurea nanocapsules (orange, dashed).

Stable aqueous dispersions of capsules were obtained after both syntheses. Size distributions were evaluated by dynamic light scattering (DLS) for 20-fold diluted dispersions, and the average diameters of the capsules were found to be 331 ± 2.3 nm for d-polyurea and 349.6 ± 2.6 nm for l-polyurea capsules (Figure 1). The size measurements were highly reproducible (evaluated in three different batches of each system) with polydispersity indexes of 0.146 ± 0.012 and 0.135 ± 0.024 for d- and l-polyurea nanocapsules, respectively. After 2.5 months, a size stability test was done for d-capsules, and the average diameter was found to be 337± 1.9 nm with a polydispersity index of 0.148 ± 0.010.

**Figure 2:**
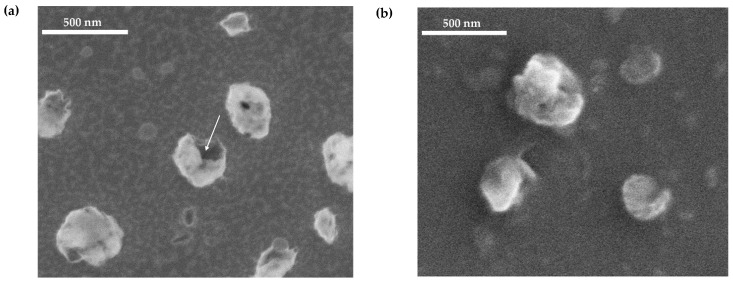
Scanning Electron Microscopy (SEM) images of: (**a**) d-chiral polyurea nanocapsules, and (**b**) l-chiral polyurea nanocapsules. Note that the capsule marked by the arrow is broken and has its hollow core exposed, which is indicative of the core-shell structure.

The morphology of the resultant polyurea nanocapsules was characterized using Scanning Electron Microscopy (SEM), and the recorded capsule size correlated well with the size distribution measured by the DLS (Figure 2). Notably, one of the d-nanocapsules captured in the SEM image shown in Figure 2 is clearly broken and its hollow core, which is indicative of the core-shell structure, can be seen.

**Figure 3:**
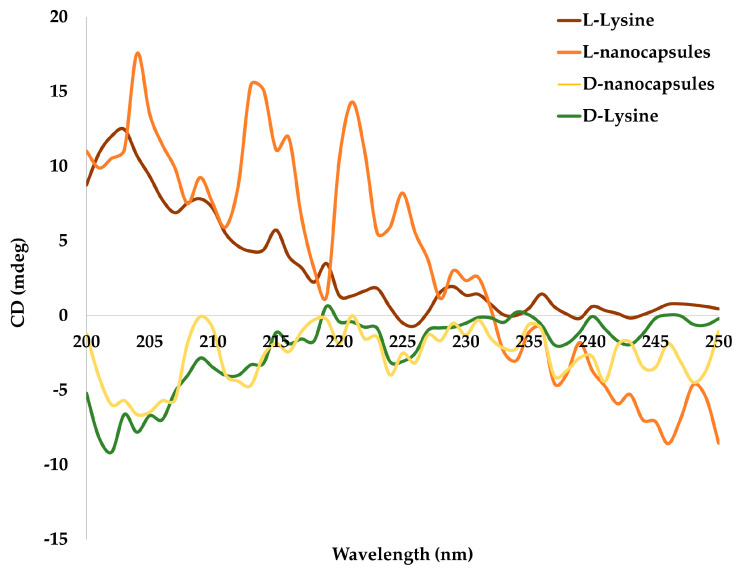
Circular dichroism spectra of free amino acid l-lysine, d-lysine, and chiral d- and l-polyurea nanocapsules. The spectra of the two types of capsules show strong opposite signals that correspond well with the spectra of free lysine amino acid enantiomers, which confirms the chirality of the capsules.

It was expected that the incorporation of the chiral amino acid (d- or l-lysine) will control the final polymeric shell configuration and, thereby, the overall chirality of the capsules. This was confirmed by the chiral optical activity of the nanocapsules (Figure 3). Circular dichroism (CD) spectroscopy revealed that the spectra of d- and l-lysine-containing nanocapsules showed strong negative and positive signals, respectively, and correlated well with their corresponding free amino acid enantiomer spectra (Figure 3).

### 2.2. DNA Adsorption onto Chiral Polyurea Nanocapsules

**Figure 4:**
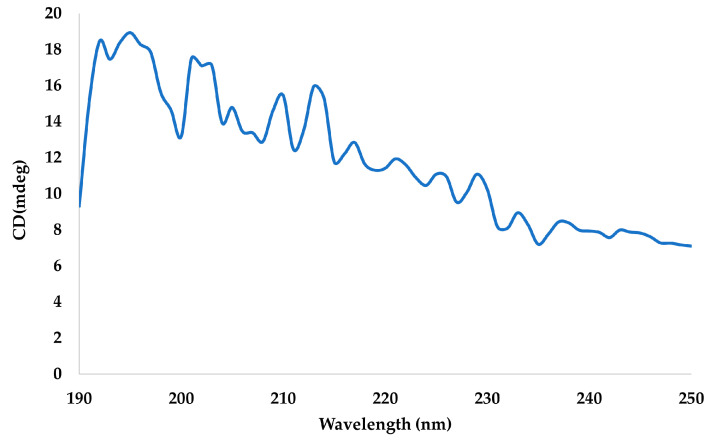
Circular dichroism spectrum of D-single stranded (ss)DNA 5′-ACA TCT TCTTCT CCC AT-3′.

As described in the experimental section, we used an oligonucleotide that is part of a gene that encodes to Actin 1 protein, with the sequence of 5′-ACA TCT TCTTCT CCC AT-3′. Like all DNA molecules from a natural origin, this sequence has a D configuration. Its chirality also was confirmed by circular dichroism analysis with a typical positive spectrum (Figure 4).

**Scheme 2:**
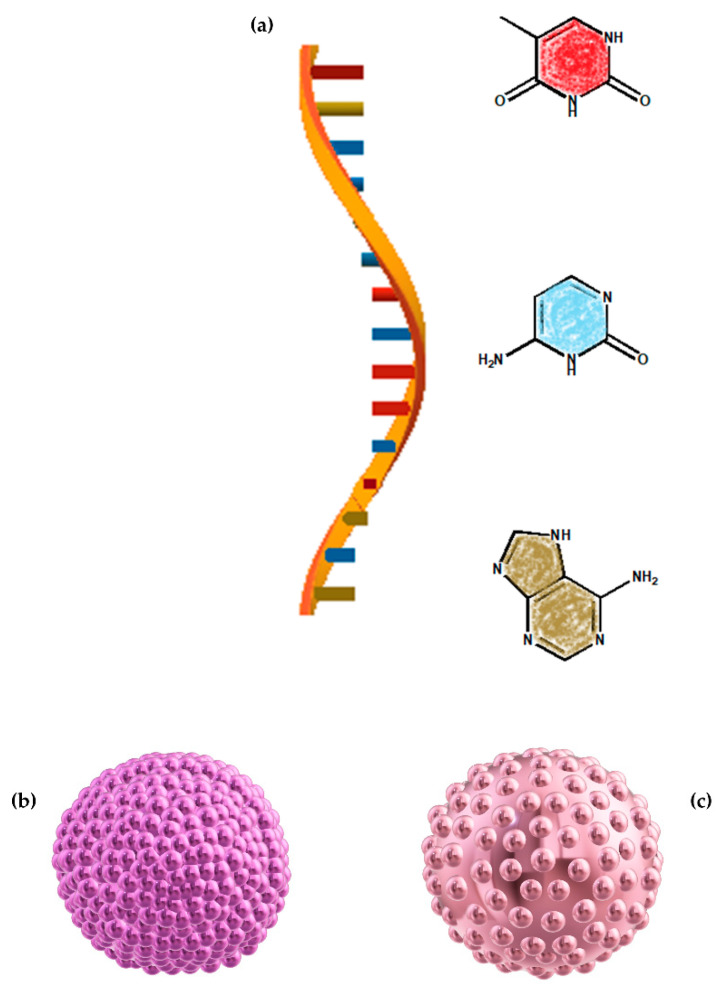
Illustrations of (**a**) 5′-ACA TCT TCTTCT CCC AT-3′ single stranded DNA structure, (**b**) Possible l-polyurea nanocapsule morphology and (**c**) Possible d-polyurea nanocapsule morphology. Please see the Discussion section for further explanation about this illustrative presentation.

A dilute single stranded DNA solution (0.25 nM final concentration) was added to d- and l-polyurea nanocapsules at a molar ratio of 27:1 (DNA: nanocapsules). The molar ratio was calculated based on the average molecular weight of the polyurea nanocapsules (determined by the dynamic light scattering (DLS)). Prior to the addition, the nanocapsules were thoroughly washed to remove polyvinyl alcohol from the surface and prevent its interference with possible stereospecific interactions. The DNA then was allowed to equilibrate with the nanocapusles, and the constructs were characterized. Scheme 2 shows the components of these systems.

**Figure 5:**
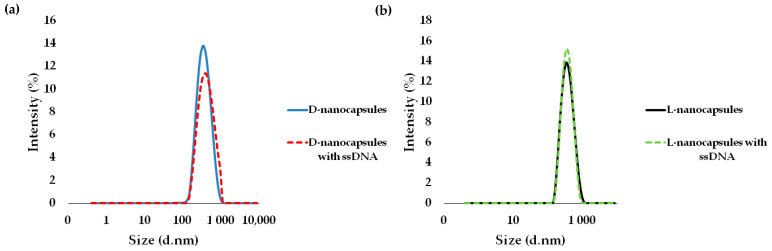
Representative size distributions of: (**a**) d-polyurea nanocapsules before (blue, solid) and after (red, dashed) the addition of dilute (ss)DNA solution and (**b**) l-polyurea nanocapsules before (black, solid) and after (green, dashed) the addition of dilute ssDNA solution. The molar ratio of DNA to nanocapsules is 27:1.

DLS measurements were performed and revealed the average size of 386.5 ± 1.8 nm and 363.9 ± 2.1 nm for the d- and l-nanocapsules, respectively, showing an increase in diameter of approximately 50 nm and 10 nm for the d- and l-nanocapsules, respectively (Figure 5).

These results show a slight adsorption of the DNA on the l-nanocapsules and a greater one on the d-nanocapsules. To ensure that the size increase occurred as a result of the DNA adsorption, the ζ-potential of the nanocapsules was measured before and after the ssDNA addition (Table 1).

**Figure 6:**
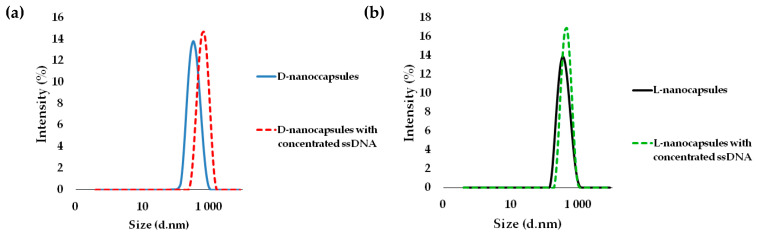
Representative size distributions of: (**a**) the d-polyurea nanocapsules before (blue, solid) and after (red, dashed) the addition of concentrated single stranded (ss)DNA solution and (**b**) l-polyurea nanocapsules before (black, solid) and after (green, dashed) the addition of concentrated ssDNA solution. The molar ratio of DNA to nanocapsules is 27 × 10^2^:1.

As shown in Table 1, the initial ζ-potentials of the d- and l-nanocapsules were very similar in value (approximately −1 mV), which can be considered neutral potential. After the addition of 0.25 nM of the ssDNA solution, both types of chiral nanocapsules became negatively charged, however, the ζ-potential values were −9 mV and −21.3 mV for l- and d-nanocapsules, respectively, which indicates a preferential adsorption of the negatively charged ssDNA [17] on the d-nanocapsule surface. To verify this effect, a concentrated solution of ssDNA (25 nM final concentration) was added to the chiral systems. The molar ratio between the ssDNA and the nanocapsules was increased 100 times. DLS measurements were performed after the system equilibration and showed a significant increase in size for the d-nanocapsules of almost 400 nm (new average size was 711 ± 2.2 nm), and a much more moderate increase in size—of approximately 100 nm—for the l-nanocapsules (new average size was 454 ± 2.6 nm) (Figure 6).

The ζ-potential of the systems was checked again and compared with the previous results (Table 2).

As shown in Table 2, after the addition of ssDNA at a higher concentration, the nanocapsules bore a greater negative charge and the ζ-potential increased to −19 mV for l-nanocapsules and to −28.5 mV for d-nanocapsules. Taken together with a fourfold larger size increase of d-nanocapsules compared with L-nanocapsules after the ssDNA addition, these results imply a preferential binding of ssDNA to d-nanocapsules.

To quantify the amount of ssDNA that gets adsorbed on the nanocapsules of the two chiral configurations, the concentration of free DNA was assessed in the 27 × 10^2^:1 ssDNA: nanocapsules chiral systems upon the removal of the capsules by filtration. UV absorption at 260 nm was measured, and the concentration of free ssDNA was determined based on the specific extinction coefficient of our oligomer sequence. Table 3 summarizes the concentrations in the filtrate after the equilibration of the systems. The adsorbed amount can be estimated from the difference between the initial and the final free DNA concentrations. It can be seen that a three times larger reduction in the free DNA concentration was observed for the d-chiral system, indicating a threefold higher adsorption on the d-nanocapsules, which is in good accordance with the nanocapsule size increase shown above.

**Scheme 3:**
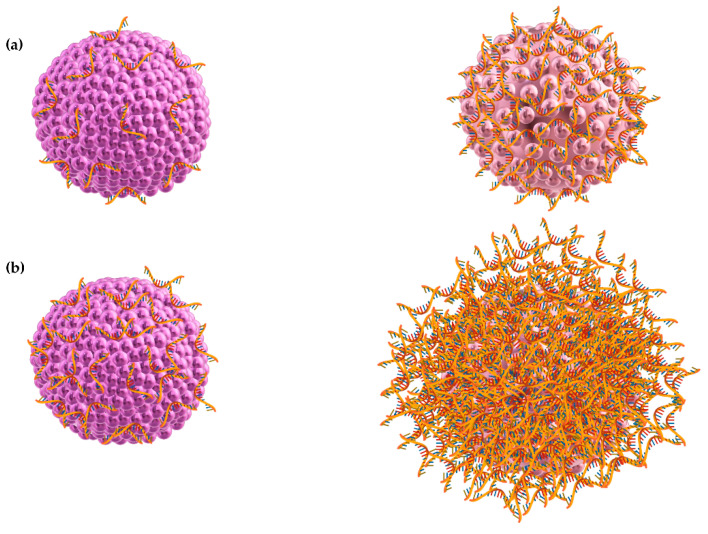
Illustrations of: (**a**) single stranded (ss)DNA adsorption from a dilute solution onto an l-nanocapsule (left) and a d-nanocapsule (right); (**b**) ssDNA adsorption from a concentrated solution onto an l-nanocapsule (left) and a d-nanocapsule (right). Chiral nanocapsules and DNA are not drawn to scale.

This confirms the preferential binding of ssDNA on the d-nanocapsule surface that has already been indicated by the greater increase in size and the more negative ζ-potential. These results infer that the chirality of the shell and the resultant capsule morphology impact DNA adsorption. An illustration of DNA interactions with chiral nanocapsules is shown in Scheme 3.

## 3. Discussion

We report on a formation of chiral hollow polyurea nanocapsules that can be used for drug delivery and theranostics. These shell structures are reproducible in size, with narrow polydispersity indexes, and have a roughly neutral surface potential. Yet, they are stable in aqueous dispersions probably owing to the steric stabilization of polymeric shells. The chirality of these nanocapsules was induced by the inclusion of d- or l-lysine amino acids in polymeric shell structures and was verified by circular dichroism spectroscopy. We then found that these nanocapsules form stereospecific supramolecular interactions with DNA. A single stranded DNA oligonucleotide with a D configuration was added to each chiral system, and its preferential adsorption on the d-polyurea nanocapsules was observed. Both a larger increase in particle size and a higher absolute value of negative ζ-potential were recorded for d-nanocapsules compared with their l-counterparts. After the initial experiment with a dilute DNA solution, a more concentrated DNA solution was added to both systems. During this experiment, a more significant size increase was achieved and an unambiguous difference in DNA adsorption between the chiral systems was demonstrated. The particle size increased by about 400 nm for d-nanocapsules and only by about 100 nm for l-nanocapsules upon the addition of DNA. Negative ζ-potentials of approximately −29 mV and −19 mV were measured for d- and l-chiral systems, respectively, upon the DNA addition (roughly 50% more negative for the d-system). The concentration of free DNA was found to be about three times lower upon its addition to the d-chiral system compared to the l-system. The particle size increase and the reduction in free DNA concentration explicitly indicate the interaction of DNA strands with the nanocapsules, while the negative ζ- potential confirms the presence of negatively charged ssDNA oligomers on the capsule surface.

**Scheme 4:**
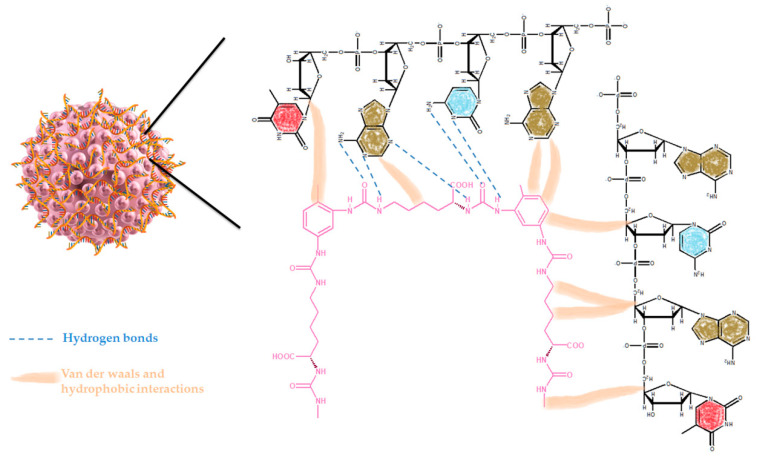
An illustration of some of the suggested interactions between the single stranded (ss)DNA oligomer and the chiral nanocapsule polyurea shell. Chiral nanocapsules and DNA are not drawn to scale.

These results suggest that the ssDNA adsorbs to the surface of the capsules with both chiral configurations; however, there are preferential supramolecular interactions with d-polyurea nanocapsules. The adsorption in general could be explained by the formation of multiple possible hydrogen bonds between the polyurea shell and the nucleobases of DNA (Scheme 4). Moreover, hydrophobic interactions are formed between the methylene groups and the carbons of the aliphatic chains and aromatic rings in polyurea and the carbons in deoxyribose sugars or the DNA backbone. Illustrations of some of the suggested interactions between ssDNA and the chiral polyurea nanocapsules are shown in Scheme 4.

**Scheme 5:**
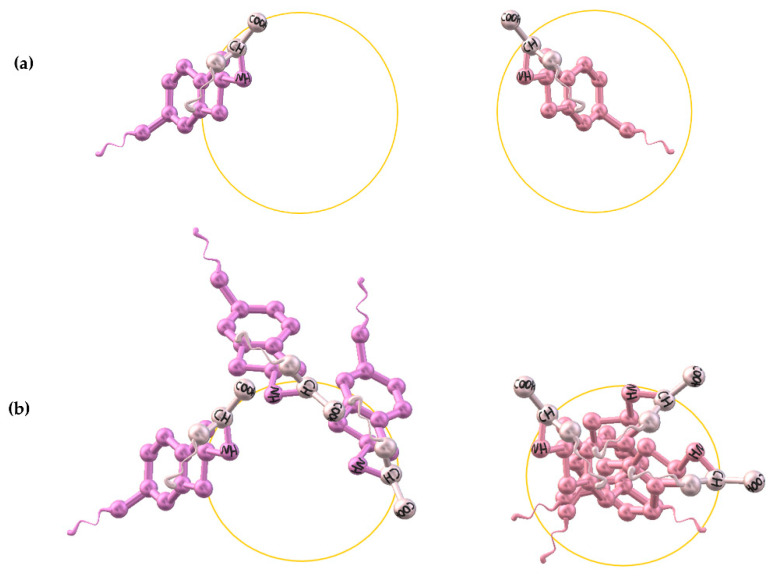
An illustration of surface polymerization direction, according to chirality, that may occur on the surface of polyurea capsules: (**a**) polymerizing d-polyurea (Right) and l-polyurea (Left) chains positioned at the same point of a spheric shell; (**b**) propagating surface polymerization of d-polyurea (Right) and l-polyurea (Left).

These aforementioned interactions are likely to occur between DNA and the capsules of both chiral configurations. However, we found a specific preferential adsorption of DNA to the d-nanocapsules. Because the chirality of the polyurea shell is determined by the chirality of the lysine unit, it seems plausible that the final surface morphology of the shell is dictated by the position of the chiral amine, and this controls the supramolecular interactions of the capsules. There is evidence in the literature that microscopic chirality can control macroscopic morphology [18,19]. If we position the 3-D models of polymerizing polyurea chains of both chiralities so that the chiral center of the lysine is at the same point on the spherical surface planes, we can assume that the direction of polymerization of the d-polyurea chains is inwards toward the particle core, whereas the direction of the polymerizing l-polyurea is outwards from the surface (Scheme 5a). Because the chiral polymerization is directionally opposite, this configuration will preserve itself throughout the capsule. This might critically affect the nanoscopic surface morphology, creating a less steric hinderance for ssDNA attachment on the d-capsules, as well as possibly exposing functional groups that have a greater affinity for ssDNA (Scheme 5b). It is noteworthy that the average hydrodynamic diameter of the l-nanocapsules is about 20 nm larger than that of the d-nanocapsules, possibly supporting the assumption of a more protruding surface morphology for the l-configuration.

To conclude, we show that the chirality of novel polymeric nanocapsules affects their supramolecular interactions with ssDNA. This unique feature may be exploited when designing new drug delivery systems for antigen/antisense gene therapy. The hollow core of these solvent-free nanocapsules can be further employed for carrying other therapeutic or diagnostic agents. The neutral surface potential, the biocompatibility, and the biodegradability of the polyurea shell ensure a low risk of in vivo toxicity [8,9,10,11].

## 4. Materials and Methods

### 4.1. Chemicals

Toluene 2,4-diisocyanate (TDI), polyvinyl alcohol (average m.w.~67,000), *n*-butyl acetate, d-lysine monohydrochloride, l-lysine monohydrochloride, and single stranded DNA (ssDNA) with the sequence of 5′-ACA TCT TCTTCT CCC AT-3′ were purchased from Sigma-Aldrich.

### 4.2. Synthesis of the Chiral Polyurea Nanocapsules

An oil phase containing 4.75 g of *n*-butyl acetate and 0.25 g of toluene 2,4-diisocyanate (TDI) was nanoemulsified with 40 g of water containing polyvinyl alcohol (PVA) 5% (*w/v*) by sonication for 50 min using a VCX-750 ultrasonic liquid processor (Sonics and Materials, Inc., Newtown, CT, USA) with an output of 750 W at 20 kHz. 0.65 g of l- or d-lysine monohydrochloride dissolved in 14.95 g of water were added to the resultant nanoemulsion, followed by heating to 35 °C for 24 h under stirring. The resulting chiral nanocapsules were washed four times with distilled water by ultracentrifugation at 25,314× *g* using an SL 40 centrifuge (Thermo Fisher Scientific, Waltham, MA, USA) to remove PVA before DNA adsorption.

### 4.3. Procedure of Adsorbing DNA from Dilute Solution onto the Chiral Nanocapsules

25 µL of 0.005 µM single stranded DNA, with the sequence of 5′-ACA TCT TCTTCT CCC AT-3′, were added to 25 µL of 1.85 × 10^−4^ µM d- or l-chiral nanocapsule dispersion (by the capsules’ molecular weights) in 450 µL of distilled water. The mixture was stirred for 2 h under ambient conditions.

### 4.4. Procedure of Adsorbing DNA from Concentrated Solution onto the Chiral Nanocapsules

25 µL of 0.5 µM single stranded DNA, with the sequence of 5′-ACA TCT TCTTCT CCC AT-3′, were added to 25 µL of 1.85 × 10^−4^ µM d- or l-chiral nanocapsule dispersion (by the capsules’ molecular weights) in 450 µL of distilled water. The mixture was stirred for 2 h under ambient conditions.

### 4.5. Characterization of Nanocapsules

#### 4.5.1. Size and ζ-Potential

The size and ζ-potential were measured by dynamic light scattering using a Zetasizer Nano model ZEN3600 (Malvern Panalytical, Malvern, UK). All measurements were performed in triplicate. Three different batches of each system were evaluated. For the size measurements, the samples were diluted 20-fold with distilled water and, for ζ-potential, 10 mM of sodium chloride solution was used at the same dilution. High resolution scanning electron microscopy images were acquired using an Extra-High Resolution Scanning Electron Microscope Magellan 400L (Thermo Fisher Scientific, Waltham, MA, USA, former FEI), equipped with a tunable diode laser (TLD) detector. The 10 µL samples were diluted 20-fold with water, as above, and placed on copper grid support films, formvar/carbon, 400 mesh, Cu (Ted Pella, Inc., Redding, CA, USA). The images were scanned at 5 kV acceleration voltages.

#### 4.5.2. Chiroptical Activity Measurements

Circular dichroism (CD) measurements were performed with an MOS-500 spectrometer (BioLogic, Seyssinet-Pariset, France). The measurements were performed over a wavelength range of 200–250 nm, using a 1 cm path length cuvette. The spectral resolution was 1 nm. The reported spectra are the results of 3–5 scans.

#### 4.5.3. Free Single Stranded DNA (ssDNA) Concentration Determination

The concentration of free single stranded (ss)DNA was estimated after filtration with Whatman FR 30/0.2 CA-S 0.2µm filters, based on the UV absorption of dispersions (diluted 20-fold with water) at 260 nm using an Ultrospec 2100 pro UV-vis spectrophotometer (Biochrom, Cambridge, UK). The concentration was calculated according to the specific extinction coefficient of the oligomer sequence (147,600 L/mol × cm).

## Figures and Tables

**Table 1:** The ζ-potential values of the chiral nanocapsules before and after single stranded (ss)DNA addition.

Type of the Nanocapsules	ζ-Potential before DNA Addition	ζ-Potential after DNA Addition ^1^
l-nanocapsules	−1.1 ± 1.3 mV	−9 ± 2.6 mV
d-nanocapsules	−1.2 ± 1.5 mV	−21.3 ± 1.4 mV

**^1^** 27:1 ssDNA: nanocapsules molar ratio.

**Table 2:** Theζ-potential values of the chiral nanocapsules before and after single stranded (ss)DNA addition.

Type of the Nanocapsules	ζ-Potential before DNA Addition	ζ-Potential after DNA Addition ^1^	ζ-Potential after Concentrated DNA Addition ^2^
l-nanocapsules	−1.1 ± 1.3 mV	−9 ± 2.6 mV	−18.8 ± 2.0 mV
d-nanocapsules	−1.2 ± 1.5 mV	−21.3 ± 1.4 mV	−28.5 ± 0.1 mV

**^1^** 27:1, **^2^** 27 × 10^2^:1 ssDNA: nanocapsules molar ratio.

**Table 3:** Single stranded (ss)DNA adsorption on nanocapsules.

System	Free DNA Concentration	Decrease in Free DNA Concentration
DNA solution	10.42 × 10^−4^ µM	-
l-nanocapsules	8.19 × 10^−4^ µM	2.23 × 10^−4^ µM
d-nanocapsules	4.16 × 10^−4^ µM	6.26 × 10^−4^ µM

## Data Availability

No additional data was generated by this study.
